# Neural Networks for Beat Perception in Musical Rhythm

**DOI:** 10.3389/fnsys.2015.00159

**Published:** 2015-11-25

**Authors:** Edward W. Large, Jorge A. Herrera, Marc J. Velasco

**Affiliations:** ^1^Department of Psychological Sciences, University of ConnecticutStorrs, CT, USA; ^2^Department of Physics, University of ConnecticutStorrs, CT, USA; ^3^Department of Music, Center for Computer Research in Music and Acoustics, Stanford UniversityStanford, CA, USA; ^4^Center for Complex Systems and Brain Sciences, Florida Atlantic UniversityBoca Raton, FL, USA

**Keywords:** beat perception, neural resonance, musical rhythm, dynamical systems, neural networks

## Abstract

Entrainment of cortical rhythms to acoustic rhythms has been hypothesized to be the neural correlate of pulse and meter perception in music. Dynamic attending theory first proposed synchronization of endogenous perceptual rhythms nearly 40 years ago, but only recently has the pivotal role of neural synchrony been demonstrated. Significant progress has since been made in understanding the role of neural oscillations and the neural structures that support synchronized responses to musical rhythm. Synchronized neural activity has been observed in auditory and motor networks, and has been linked with attentional allocation and movement coordination. Here we describe a neurodynamic model that shows how self-organization of oscillations in interacting sensory and motor networks could be responsible for the formation of the pulse percept in complex rhythms. In a pulse synchronization study, we test the model's key prediction that pulse can be perceived at a frequency for which no spectral energy is present in the amplitude envelope of the acoustic rhythm. The result shows that participants perceive the pulse at the theoretically predicted frequency. This model is one of the few consistent with neurophysiological evidence on the role of neural oscillation, and it explains a phenomenon that other computational models fail to explain. Because it is based on a canonical model, the predictions hold for an entire family of dynamical systems, not only a specific one. Thus, this model provides a theoretical link between oscillatory neurodynamics and the induction of pulse and meter in musical rhythm.

## 1. Perception of pulse and meter in musical rhythms

The sounds that humans use for communication are temporally structured sequences of events, such as musical notes or speech syllables. Rhythm refers to the pattern of timing and stress in the amplitude envelope of an acoustic sequence. Musical rhythms are usually perceived to have a pulse, or basic beat, in the approximate frequency range 0.5–4 Hz (London, 2004). Meter is a perceived temporal structure that includes the pulse frequency, as well as slower beat frequencies that accent pulse cycles (< 2 Hz), and higher beat frequencies that subdivide the pulse (4–8 Hz) (Lerdahl and Jackendoff, 1983; London, 2004). Neural resonance theory (Large and Snyder, 2009) hypothesizes that pulse and meter correspond to neural rhythms that synchronize with acoustic rhythms, influencing temporal expectancy, attention, and movement coordination. Theoretical approaches to understanding perceived structure have been based on neurodynamic models of neural oscillation (for a review, see Large, 2008). However, only recently has a clear picture begun to emerge regarding synchronization of neural oscillations and the neural structures that support responses to musical rhythm.

In this paper, we summarize current knowledge about the synchronization of neural rhythms to musical rhythms and outline a neurodynamic model of pulse perception based on entrainment of neural oscillation. First, in §2, we present a brief overview of the main theories and experimental findings related to musical pulse and meter. We discuss the potential function of neural oscillations in establishing the perceived temporal structure of complex musical rhythms. In §3, we sketch a neurodynamic model of pulse perception based on the interaction between oscillatory neural networks. The model incorporates the basic findings of the past 20 or so years and makes a key prediction about the formation of the pulse percept. In §4, we evaluate the fundamental prediction of the theory, that perceived temporal structures may correspond to frequencies that are not physically present in the amplitude envelope. Finally, in §5, we discuss remaining important questions regarding the link between pulse perception and neural resonance.

## 2. Neural resonance to musical rhythms

**Figure 1:**
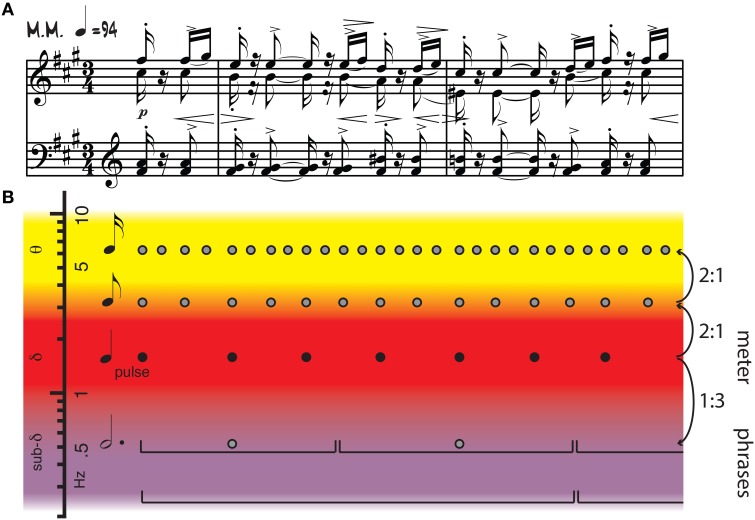
**(A)** Piano score of Isaac Albeniz's Iberia II, Triana. **(B)** Annotation showing pulse (black dots), metrical structure (all dots), and phrasing (brackets). Pulse frequencies overlap with cortical oscillations in the delta range, while other metrical frequencies extend into theta and sub-delta ranges. Frequency relationships among metrical levels include harmonics, subharmonics, and other integer ratios. Adapted from Large (2014).

Neural population rhythms—as observed in the local field potential (LFP), the electroencephalogram (EEG), and the magnetoencephalogram (MEG)—are cyclical fluctuations of baseline neuronal activity that can be observed in neocortical and thalamic regions of the brain (Steriade et al., 1993; Buzsáki and Draguhn, 2004; Slézia et al., 2011). Thalamocortical oscillations exhibit 1∕*f* frequency spectra with peaks in specific frequency bands, including delta (~ 1–4 Hz), theta (~ 4–8 Hz), beta (~ 13–30 Hz), and gamma (~ 30–70 Hz) (Buzsáki, 2006). The frequency range of musical pulse (London, 2004) corresponds nicely with the delta band, while faster metrical frequencies fall within the theta band, and slower metrical frequencies occupy the sub-delta range (Musacchia et al., 2014, see Figure 1). Jones originally hypothesized that endogenous perceptual rhythms synchronize with temporally structured sequences, generating expectancies for future events (Jones, 1976). Dynamic attending theory (DAT; Jones, 1976; Jones and Boltz, 1989; Large and Jones, 1999) addressed the issue of how neural rhythms may be exploited by an organism to enable attentional coordination with the dynamic external world (for a review, see Jones, 2008). The sensory-motor theory of rhythm perception (Todd, 1994, 1995; Todd and Lee, 2015) hypothesized that rhythm and pulse perception involve a sensory representation of the input as well as a motor representation of the body. A related theoretical framework that accounts for the predictive role of the motor system has recently been proposed (Schubotz, 2007). Converging lines of investigation have provided evidence supporting both theoretical perspectives, and suggest that interaction between sensory and motor regions of the brain may provide a mechanism for predicting sequence timing (Schroeder et al., 2010). In this paper, we further develop a neural resonance theory of rhythm perception, hypothesizing that oscillatory interactions between sensory and motor areas are sufficient to give rise to percepts of pulse and meter in complex musical rhythms (Large and Snyder, 2009).

Behavioral evidence for dynamic attending in adults and infants comes from time discrimination and pitch discrimination studies that revealed an advantage for auditory sequences that induce a metric percept, using both sensitivity and reaction time measures (Yee et al., 1994; McAuley and Kidd, 1995; Jones and Yee, 1997; Large and Jones, 1999; Barnes and Jones, 2000; Jones and McAuley, 2005; Bergeson and Trehub, 2006; Jones et al., 2006). Such findings are consistent with early-developing predispositions for temporal regularity, and a preference for more stable temporal organizations, as predicted by nonlinear resonance (Large, 2008). In support of sensory-motor theory, a fundamental sound-movement interaction was demonstrated in infants and adults such that vestibular stimulation can influence whether an ambiguous pattern is perceived in duple or triple meter (Phillips-Silver and Trainor, 2005, 2007). The top-down influence of motor networks on sensory representations has been recently studied, showing how the former can “sharpen” the temporal selection of auditory information (Morillon et al., 2014, 2015). In addition, the finding of perceptual narrowing in infants demonstrated that meter perception is plastic and depends upon musical enculturation (Hannon and Trehub, 2005).

**Figure 2:**
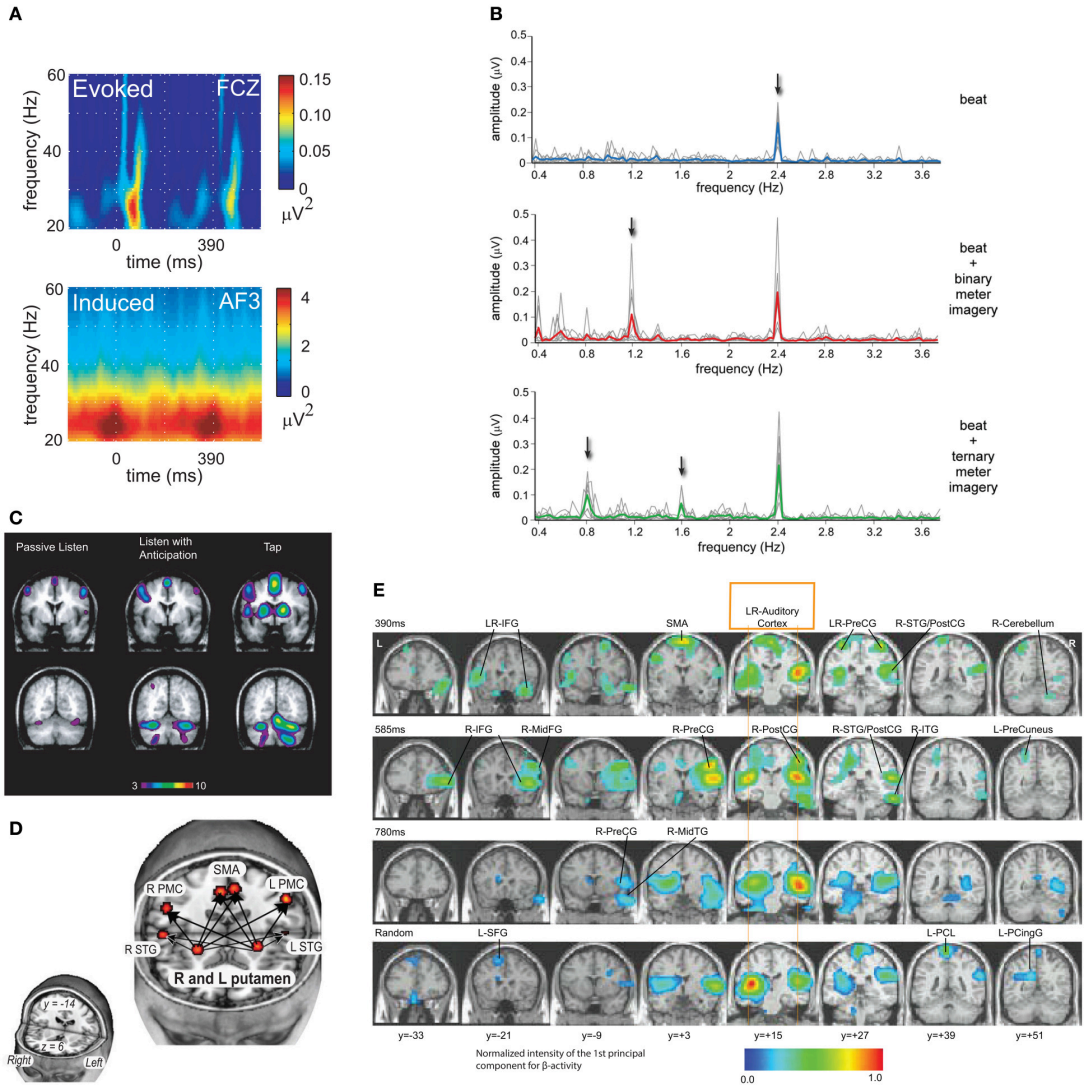
**(A)** EEG revealed synchronized fluctuations in induced beta- and gamma-band power that anticipated tone onsets, were sensitive to intensity accents, and persisted when expected tones were omitted. Evoked activity occurred after tone onsets and were strongly diminished after tone omissions (Snyder and Large, 2005; reprinted with permission). **(B)** A periodic rhythm elicited a steady-state evoked potential (SS-EP) at the stimulus repetition frequency, and meter imagery elicited subharmonic resonances corresponding to the metric interpretation of this periodic rhythm (Nozaradan et al., 2011; reprinted with permission). **(C)** fMRI showed that listening to musical rhythms recruits both auditory and motor areas of the brain even in perception tasks without a motor component (Chen et al., 2008a; reprinted with permission). **(D)** Functional connectivity analysis revealed a cortico-subcortical network including the putamen, SMA, and PMC under conditions that may require internal generation of the pulse (Grahn and Rowe, 2009; reprinted with permission). **(E)** MEG revealed oscillatory interactions in a striato-thalamo-cortical network (Fujioka et al., 2012; reprinted with permission).

EEG and MEG studies have directly tested the role of endogenous oscillation. One EEG study revealed that fluctuations in induced beta- and gamma-band power synchronized with periodic and metrical rhythms, revealing both sensory-driven and anticipatory responses to tones (Snyder and Large, 2005; Figure 2A). A related MEG study also found sensory and anticipatory responses in the beta- and gamma-band (Fujioka et al., 2009). When subjects were instructed to impose a subjective meter on a periodic stimulus, subharmonic responses were observed in induced beta band MEG activity, closely resembling those produced by physical accents (Iversen et al., 2009). One EEG study reported synchronization of delta rhythms, phasic responses in theta, and augmented phase synchronization throughout the beta/gamma range, modulated by the stimulus periodicity (Will and Berg, 2007). Another study revealed entrainment of the delta rhythm to the onset of target tones, and reaction times that correlated with the phase of the delta band oscillation at target onset, directly supporting the role of neural oscillation in attending (Stefanics et al., 2010). The steady-state evoked potential (SS-EP) technique revealed that a periodic rhythm elicited a sustained response in the delta band, and meter imagery elicited an additional subharmonic resonance corresponding to the metric interpretation (Nozaradan et al., 2011; Figure 2B). Another study showed that complex rhythms elicited multiple SS-EPs in the EEG spectrum at frequencies corresponding to the rhythmic pattern envelope, and the amplitude of the SS-EPs at pulse and meter frequencies was selectively enhanced, suggesting a role for neural oscillations in pulse and meter induction (Nozaradan et al., 2012). Thus, delta, beta and gamma band responses to auditory rhythms observed in both in EEG and MEG in humans have confirmed predictions of dynamic attending theory. Given the role of beta in motor processing and long-range intra-cortical interaction, these findings are consistent with the idea that the motor system influences the perception of sound, even in the absence of overt movement.

Functional magnetic resonance imaging (fMRI) studies of musical rhythm perception have clearly demonstrated that listening to musical rhythms recruits both auditory and motor areas of the brain (Chen et al., 2008a; Figure 2C). A functionally connected network is implicated in extracting higher-order features of a rhythm's temporal structure, with the dorsal premotor cortex mediating auditory-motor interactions, and functional coupling of auditory-motor networks observed even in perception tasks without a motor component (Chen et al., 2008b). Such studies have implicated the basal ganglia (Grahn and Rowe, 2009), cerebellum and supplementary motor area (Chen et al., 2008a; Bengtsson et al., 2009), as well as the dorsal premotor cortex and right frontal lobe (Bengtsson et al., 2009). Functional connectivity analysis implicates a cortico-subcortical network including the putamen, SMA, and PMC for the analysis of temporal sequences, especially under conditions that may require internal generation of the pulse (Grahn and Rowe, 2009; Figure 2D). Basal ganglia may play an important functional role in the formation of the pulse percept (Chapin et al., 2010; Kung et al., 2013) or pulse prediction (Grahn and Rowe, 2013). Although functional imaging cannot resolve rhythmic time scales, MEG source analysis has revealed beta band interactions in auditory and motor networks during musical rhythm processing, implying oscillatory interactions among auditory and motor cortices as well as the cerebellum, thalamus, and parahippocampal gyrus (Fujioka et al., 2012; Figure 2E). Synchronization of beta suppression with the auditory input (Fujioka et al., 2012) and the spatial overlap of the beta network with the striato-thalamo-cortical network implicated by functional imaging suggests that the mechanism of pulse and meter perception is fundamentally oscillatory. Moreover, in macaque monkeys, beta- and gamma-band oscillations measured using LFPs during synchronization-continuation tasks suggest differential roles in rhythmic tapping versus stimulus processing (Bartolo et al., 2014). Observations of beta oscillations in basal ganglia during synchronization and continuation further support their role in the striato-thalamocortical circuit during control of rhythm (Bartolo and Merchant, 2015; for a recent review, see Merchant et al., 2015a). Interaction between sensory and motor regions of the brain has been found not only when listening to music, but also in speech perception and production (Rauschecker and Scott, 2009).

**Figure 3:**
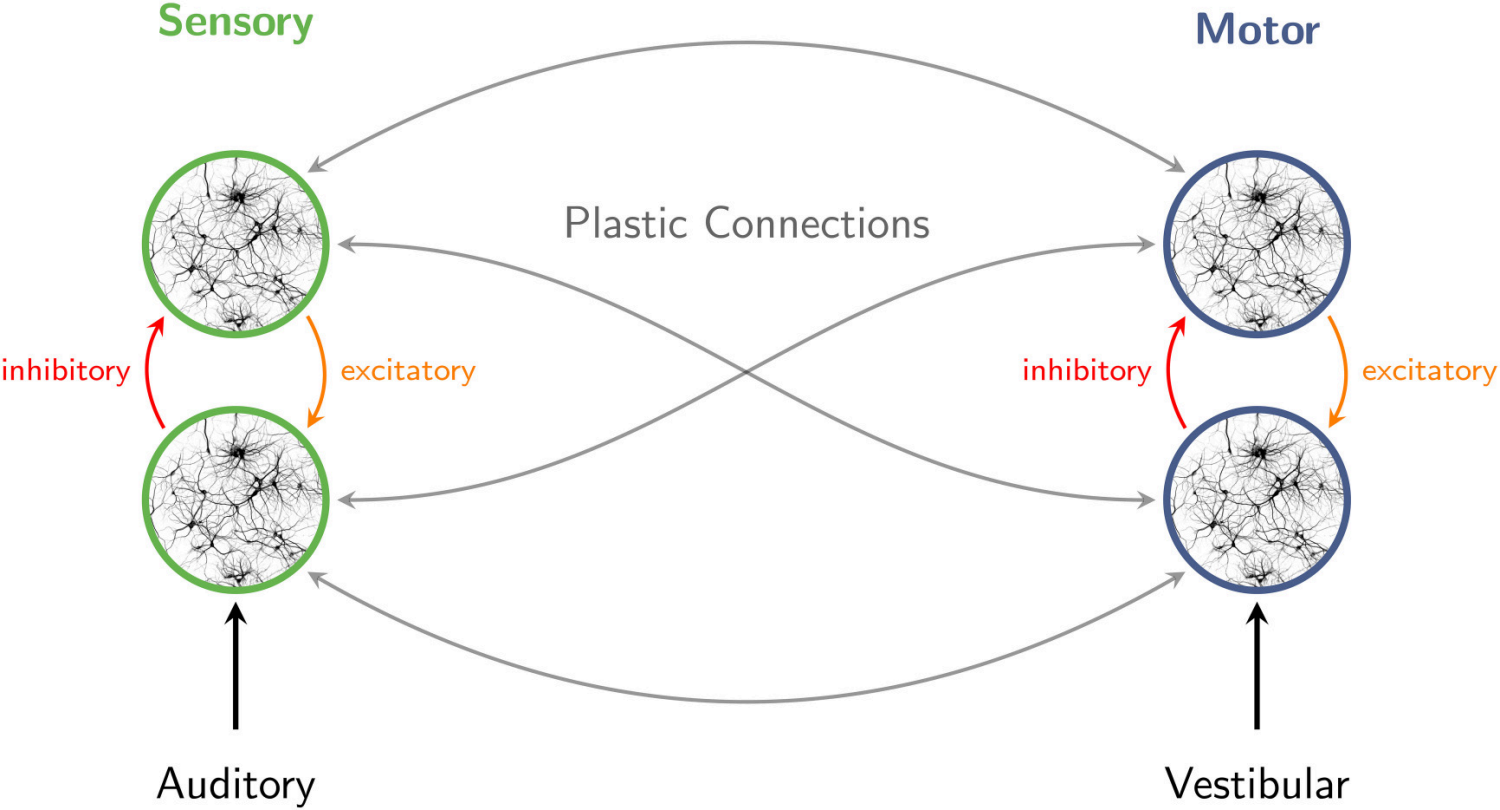
**Interaction of excitatory and inhibitory neuronal populations gives rise to population rhythms in sensory and motor networks**. When sensory stimuli are presented in a periodic pattern, auditory cortical oscillations entrain to the structure of the stimulus stream. The perception of pulse and meter in music also involves broadly distributed motor systems. Auditory-motor coupling is reciprocal, as vestibular stimulation can influence auditory rhythm perception. Connections within and between sensory and motor systems are assumed to be plastic.

A summary of this scenario is outlined in Figure 3. Interaction of excitatory and inhibitory neuronal populations gives rise to population rhythms throughout the brain (Brunel, 2003; Börgers and Kopell, 2003; Buzsáki and Draguhn, 2004; Stefanescu and Jirsa, 2008), including sensory and motor networks. When sensory stimuli are presented in a periodic pattern, ambient delta-band oscillations entrain to the structure of the stimulus stream (Will and Berg, 2007; Nozaradan et al., 2011, 2012). Fluctuations in beta- and gamma-band rhythms synchronize as well, consistent with an oscillatory hierarchy in auditory cortex (Lakatos et al., 2005). Neuronal entrainment emerges rapidly and facilitates behavioral responses (Stefanics et al., 2010). The perception of pulse and meter involves broadly distributed motor systems (Chen et al., 2008a; Grahn and Rowe, 2009, 2013; Kung et al., 2013) which respond with synchronized fluctuations in gamma- and beta-band amplitude (Fujioka et al., 2012), enabling coordination of perception and rhythmic movements with musical rhythms (Chen et al., 2008b; Nozaradan et al., 2013). Auditory-motor coupling is reciprocal (Phillips-Silver and Trainor, 2005, 2007), and connections within and between sensory and motor systems are assumed to be plastic (Hannon and Trehub, 2005).

## 3. Oscillatory network interactions could give rise to musical pulse perception

A fundamental challenge to understanding a complex neural system such as the one just described lies in the integration of data from multiple modalities and multiple levels of observation into a coherent systems model of perception, attention and behavior. Musical rhythm provides a unique opportunity in this regard, because acoustic signals are thought to drive entrainment of neuronal population oscillations. Nonlinear oscillation in general, and neural oscillation in particular, have been widely studied in the mathematical and physical sciences over the past 50 years, putting us in a unique position to link levels of observation from spiking neurons to population dynamics and from population dynamics to perception and behavior, because sophisticated mathematical tools are available for understanding the behavior of such systems. Models of neuronal oscillations arising from the interaction of excitatory and inhibitory populations of neurons can be used to study mechanisms of neural oscillation (Whittington et al., 2000), with implications for many different aspects of neural information processing (Wilson and Cowan, 1973; Hoppensteadt and Izhikevich, 1999; Varela et al., 2001; Koepsell et al., 2010; Ainsworth et al., 2012). Models may be driven with external input (Brunel, 2003; Large et al., 2010), may include synaptic plasticity (Hoppensteadt and Izhikevich, 1996b; Brunel, 2003), and predict a surprisingly rich repertoire of behaviors, including steady states, Hopf bifurcations, double limit cycle bifurcations, bursting, and chaotic dynamics (Brunel, 2000; Stefanescu and Jirsa, 2008; Ledoux and Brunel, 2011).

Mathematical models can be useful in linking oscillation of high dimensional neuronal populations with lower dimensional population-level models that capture much of the behavioral richness observed in high dimensional systems (Wilson and Cowan, 1973; Stefanescu and Jirsa, 2008), and are amenable to theoretical and computational analysis (Aronson et al., 1990; Hoppensteadt and Izhikevich, 1996a). We have proposed one such population-level model (1; Large et al., 2010) to describe the dynamics of networks of neural oscillators with different natural frequencies responding to external stimulation:

(1) $$\begin{array}{l} \tau_{i}\frac{dx_{i}}{dt}=f_{i}(x_{i},y_{i},\lambda )+\epsilon p_{i}(x_{1},y_{1},\ldots ,x_{n},y_{n},s(t),\lambda ,\epsilon )\\ \tau_{i}\frac{dy_{i}}{dt}=g_{i}(x_{i},y_{i},\lambda )+\epsilon q_{i}(x_{1},y_{1},\ldots ,x_{n},y_{n},s(t),\lambda ,\epsilon ) \end{array}$$

Here, the variables *x*_*i*_ and *y*_*i*_ represent excitatory and inhibitory activities in the *i*th neuronal population, respectively. The nonlinear functions *f*_*i*_ and *g*_*i*_ describe the intrinsic (uncoupled) dynamics of the excitatory and inhibitory subpopulations, and *p*_*i*_ and *q*_*i*_ describe interactions between neuronal subpopulations with external input *s*(*t*). λ is a set of model parameters and τ_*i*_ is varied to create a frequency gradient as is found in many parts of the auditory system (Langner, 1992). ϵ is a small number that represents weak interaction (Hoppensteadt and Izhikevich, 1996a; Large et al., 2010).

This very general model implies a set of generic predictions about emergent neuronal oscillations under the influence of time-varying external input (e.g., musical rhythms) that hold under a broad set of assumptions (Hoppensteadt and Izhikevich, 1996a; Large et al., 2010). Equation (2) describes a canonical gradient frequency neural oscillator network derived from Equation (1) using normal form theory (Large et al., 2010), that has been used to model entrainment of perception, attention and behavior to rhythmic stimuli (Large, 2010).

(2) $$\begin{array}{l} \tau_{i}\frac{dz_{i}}{dt}=z_{i}(\alpha +\text{i}2\pi +\beta_{1}{\left|z_{i}\right|}^{2}+\epsilon \beta_{2}{\left|z_{i}\right|}^{4}+\ldots )\\ \;+\sum_{j\neq i}^{n}c_{ij}P(\epsilon ,z_{j})A(\epsilon ,\bar{z_{i}})+b_{i}s(t) \end{array}$$

**Figure 4:**
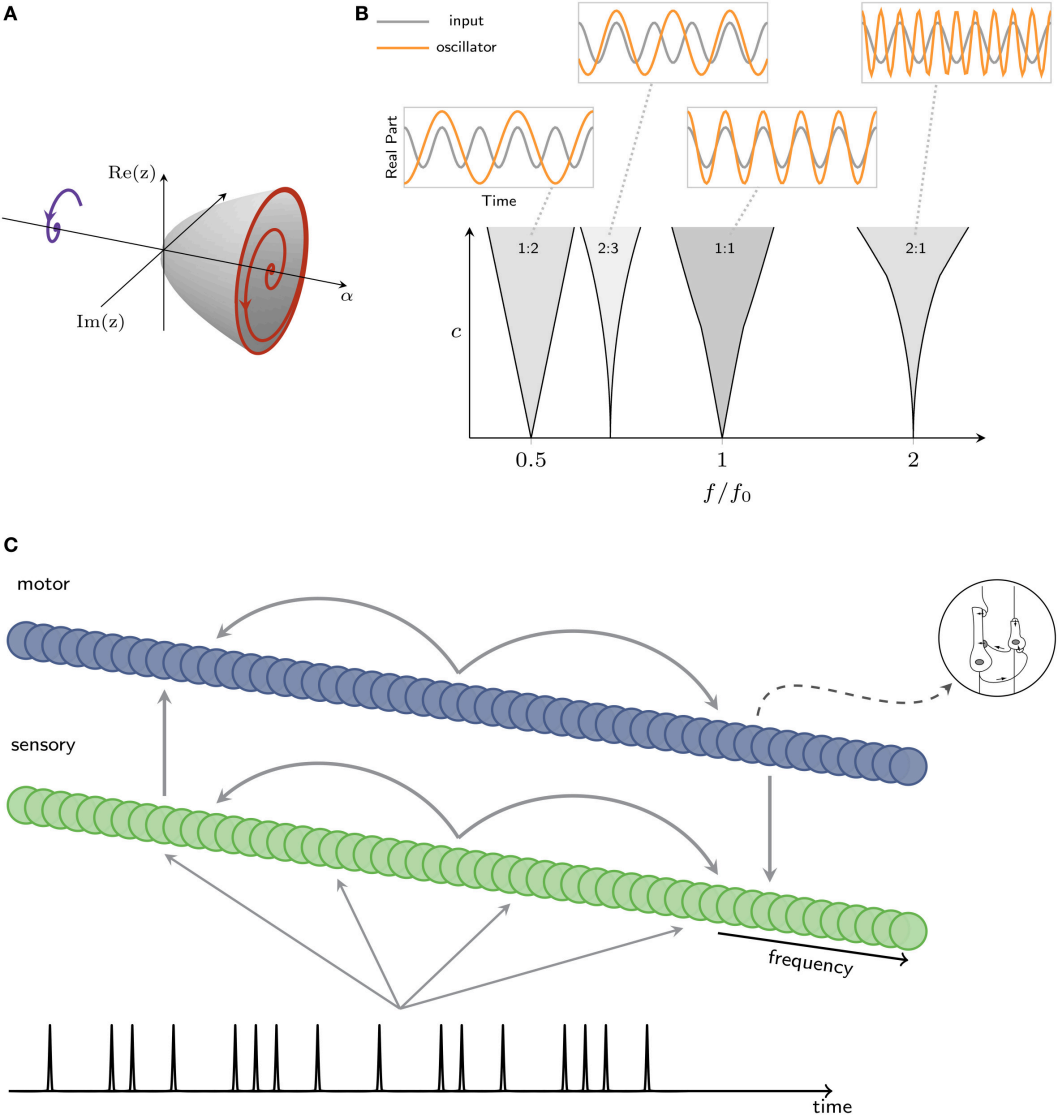
**(A)** Intrinsic oscillatory dynamics can take the form of a Hopf bifurcation, where a value α = 0 is the critical point, between damped (left) and spontaneous (right) oscillation. **(B)** Mode locking in the canonical model. Within each resonance region (shaded), the canonical model mode-locks to input at the ratio shown in figure (*c*: coupling strength, *f*: oscillator's intrinsic frequency, *f*_0_: input frequency). Insets show the inputs and traces produced by a canonical model. **(C)** Architecture of a model that captures interacting oscillatory dynamics in sensory and motor networks. A rhythm is input to a sensory network; sensory and motor networks are reciprocally connected, providing input to one another.

Here the roman *i* denotes the imaginary unit and *z*_*i*_ is the complex-valued state variable for the *i*th neural oscillator whose real and imaginary parts can be thought of as the activation of the excitatory and inhibitory subpopulations, respectively. The *i*th oscillator's natural frequency is given by *f*_*i*_ = 1∕τ_*i*_. The β's are nonlinear damping parameters, and the complete expansion of intrinsic terms describes a fully saturating nonlinearity (Murdock, 2003; Large, 2010). The parameter α controls the system's intrinsic behavior. α = 0 is the critical point, above which the system exhibits spontaneous oscillation and below which damped oscillation, through an Andorov-Hopf bifurcation, illustrated in Figure 4A. The interaction of the external signal with the intrinsic oscillatory dynamics makes the key predictions in this model.

Nonlinear stimulus coupling predicts mode-locked responses of neural oscillators to the stimulus. Mode-locking is a generalization of phase-locking in which a periodic stimulus interacts with an intrinsic oscillatory dynamics of a neuron or neural circuit, causing *k* cycles of an oscillation to lock to *m* cycles of the stimulus, where *k* and *m* are integers, as shown in Figure 4B (for phase-locking, *k* = *m* = 1). Mode-locking predicts neural responses at harmonics, subharmonics, integer ratios, and combination frequencies of those present in a given rhythmic stimulus. Mode-locking in the canonical model is captured using a full expansion of resonant monomials (Large et al., 2010) which is expressed as a “passive” nonlinear function P of input from another oscillator, *z*_*j*_, multiplied by an “active” nonlinear function A of the current state, *z*_*i*_:

(3) $$P(\epsilon ,z)=z+\;\sqrt{\epsilon }z^{2}+\epsilon z^{3}+\epsilon \sqrt{\epsilon }z^{4}+\ldots =\frac{z}{1-\sqrt{\epsilon }z}$$

(4) $$A(\epsilon ,\bar{z})=1+\sqrt{\epsilon }\bar{z}+\epsilon {\bar{z}}^{2}+\epsilon \sqrt{\epsilon }{\bar{z}}^{3}+\ldots =\frac{1}{1-\sqrt{\epsilon }\bar{z}}$$

We use two gradient frequency networks to model the functional coupling of auditory-motor networks observed in perception tasks without a motor component (Chen et al., 2008a; Velasco and Large, 2011). The sensory network is intended to capture auditory cortical entrainment, while the motor network is intended to capture the dynamics of a broadly distributed network including basal ganglia and cortical areas. As illustrated in Figure 4C, the sensory network takes a rhythmic input, sends output to a motor network, and the motor network send input back to the sensory network (for a detailed description, see Velasco and Large, 2011). Connections within and between networks are assumed to be plastic and tuned by musical enculturation (cf., Hannon and Trehub, 2005; Large, 2010; Velasco and Large, 2011).

**Figure 5:**
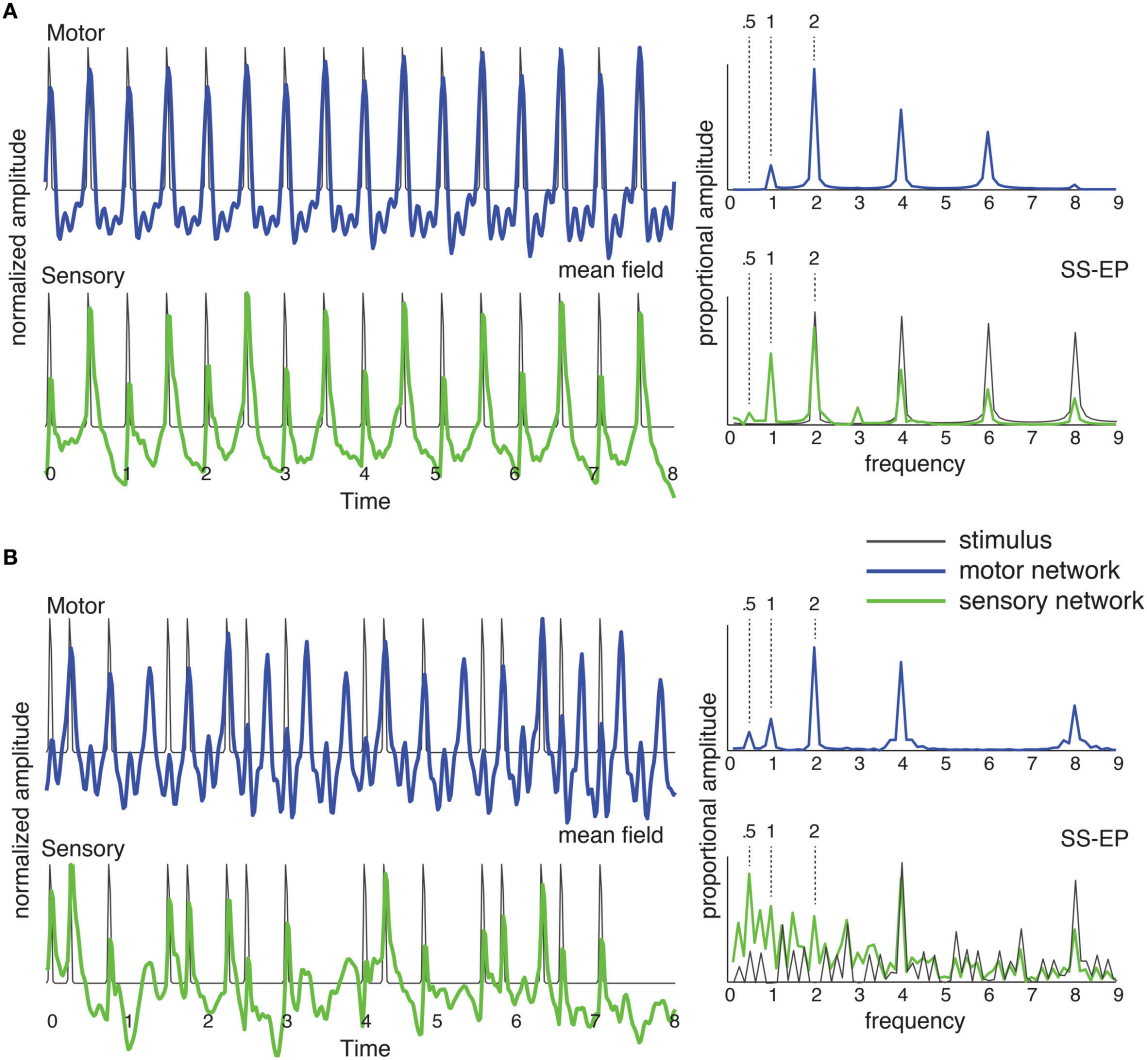
**The network of Figure 4C was stimulated with (A) an isochronous rhythm and (B) a “missing pulse” rhythm**. Output of the sensory network is in green and output of the motor network is in blue. The mean field time series (left) was obtained by summing the output of all the oscillators in the network over time. The SS-EP is obtained by Fourier analysis (DFT) of the mean field time series. DFT of the stimulus envelope is shown in black on the sensory SS-EP axis.

Although the model makes only very general assumptions regarding underlying neural structures (e.g., Chen et al., 2008a), it makes strong commitments about the oscillatory dynamics of auditory-motor interactions (Will and Berg, 2007; Fujioka et al., 2012; Nozaradan et al., 2013). The sensory oscillators are tuned to operate near a Hopf bifurcation (Hoppensteadt and Izhikevich, 1997), as shown in Figure 4A; the motor oscillators are tuned to operate near a double limit cycle bifurcation (Izhikevich, 2000; Velasco and Large, 2011). The double limit cycle regime of the motor network means that the model can capture synchronization-continuation behavior, continuing to produce rhythmic behavior after the stimulus ceases (Wing and Kristofferson, 1973a,b). Here, however, we are interested in evaluating predictions about synchronization (Velasco and Large, 2011). To predict mean field time series as observed in EEG recordings (e.g., Will and Berg, 2007; Stefanics et al., 2010), we sum the output of all oscillators in each network (Figure 5, left). To predict steady state evoked potentials (SS-EPs, e.g., Berens and Velasco, 2009) we take a frequency analysis (DFT) of the mean field (Figure 5, right).

As shown in Figure 5A, for a periodic stimulus, both sensory and motor networks produce synchronized oscillations at the pulse frequency, and generate harmonics (Repp, 2008) and subharmonics (Vos, 1973; Bolton, 1894; Nozaradan et al., 2011). In the case of a complex rhythm, however, it becomes clear that the two networks are doing something quite different from one another. The mean field time trace for the sensory network represents the input rhythm rather faithfully, producing well-defined pulses at input event times. By contrast, the motor network entrains at the pulse frequency. The rhythm itself contains no energy at the pulse frequency (or its second subharmonic; DFT in Figure 5B, SS-EP, solid black), however, in the motor network the strongest response is found at the pulse frequency. In other words, the development of the pulse percept depends on the interaction of these two oscillatory systems.

This predicts that an oscillatory network interaction can lead to spontaneous pulse induction in complex rhythms—even in the most extreme case of a rhythm for which there is no energy at the pulse frequency. Thus, the theoretical prediction is that pulse may be perceived at a frequency that is not physically present in the rhythmic stimulus (Large, 2010; Velasco and Large, 2011). The prediction for an isochronous rhythm (Figure 5A) is consistent with observations of subharmonic resonance in EEG (Nozaradan et al., 2011), however, in that EEG study subharmonic resonance was observed only when subjects were instructed to imagine it. The behavior of the sensory network in response to the complex rhythm (Figure 5B) is consistent with the observation of the enhancement of metrical frequencies in the SS-EP (Nozaradan et al., 2012). However, the prediction of network interaction goes further in that the motor network produces metrical frequencies that are completely absent from the rhythm. This is a strong prediction that has never been tested.

This points up a critical issue in the interpretation of most of the empirical studies that have been conducted to date. The problem that arises in the theoretical interpretation of synchronized periodic movements or population-level neural recordings is that of linking observations of synchrony with intrinsic dynamics of emergent population oscillations (Whittington et al., 2000). EEG oscillations, for example, represent a remote consequence of periodic activity in the area of the brain between recording electrodes and a reference electrode. To generate a signal distinct from background noise, this activity must be manifest in a large enough proportion of neurons proximal to the electrode, implying a certain degree of synchronization among neurons. However, there are at least two distinct mechanisms by which neuronal synchrony can occur: it can be a consequence of an emergent population oscillation within the network of cells under the electrode—with its own intrinsic dynamics—or it could be merely a consequence of a common rhythmic input (Whittington et al., 2000). In neural resonance theory it is postulated that both are present, i.e., a common rhythmic input entrains an emergent population oscillation. In this case, interpretation of a synchronized neural responses is especially tricky, because a synchronized response itself does not imply interaction of the input with an intrinsic oscillatory dynamics. However, demonstration of a pulse percept at a frequency that is not physically present in a stimulus rhythm would directly implicate intrinsic oscillatory dynamics in the perception of pulse.

## 4. Does rhythmic input interact with intrinsic dynamics to form the pulse percept?

Thus, the fundamental prediction to be tested is whether people will perceive a pulse frequency that is not present in a rhythmic stimulus. This is a strong prediction, because if pulse perception could be experimentally observed at a missing frequency, it would rule out the interpretation of synchronization due merely to a common input frequency, and equivalently models of pulse induction based on linear resonance. On the other hand, if a predicted missing pulse were not observed, it would falsify the hypothesis that pulse induction occurs due interaction between a rhythmic stimulus and the intrinsic dynamics of endogenous oscillatory networks.

**Figure 6:**
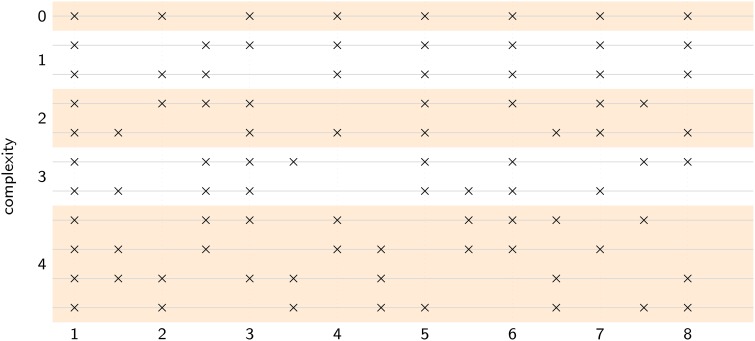
**Each stimulus pattern contained eight events distributed over eight pulse cycles**. Complexity was varied by manipulating the number of events that fell in phase versus anti-phase with the intended pulse. At complexity level 0, all eight events were in-phase, at complexity level 1, one event was anti-phase, at complexity level 2, two events were anti-phase, and so on. Level 4 was the most complex, with four events in-phase and four events anti-phase. One isochronous control (level 0), two level 1 patterns, two level 2 patterns, two level 3 patterns, and four level 4 patterns were used, for a total of 11 rhythms.

Pulse is operationally defined as the frequency at which a listener coordinates a periodic movement with a complex rhythm (Large, 2008). Thus, we asked participants to listen to eleven rhythms ranging from isochronous to highly complex, where the most complex rhythms contained no spectral energy at the pulse frequency predicted by the model, as shown in Figure 6. Participants were instructed to listen to each rhythm until they heard a steady pulse, and then tap along with the rhythm at that rate. The fourteen participants were primarily undergraduates from Florida Atlantic University, who received partial credit in fulfillment of undergraduate psychology courses, one participant was a volunteer, and none were professional musicians (8 males, 6 females; mean age 25.3; mean years of musical training: 3.2). All participants provided informed consent, following guidelines set by the Institutional Review Board of Florida Atlantic University.

Rhythms were presented at five levels of complexity (0–4) and at five different tempi (i.e., pulse frequencies): 2.28 Hz (420 ms), 2.17 Hz (460 ms), 2 Hz (500 ms), 1.85 Hz (540 ms), and 1.72 Hz (580 ms). The location of acoustic events on a metric grid was used to manipulate the level of rhythmic complexity (i.e., syncopation, see Figure 6). Counting quarter-note beats as “strong” and eighth-note subdivisions as “weak” (Lerdahl and Jackendoff, 1983), the level of syncopation was varied by manipulating the number of events that fell on weak beats, as illustrated in Figure 6. Weak beats (subdivisions) can also be thought of as existing in anti-phase relation to the in-phase strong beats. At complexity level 0, all eight events fell on strong beats, at complexity level 1, one event fell on a weak beat and seven events fell on strong beats, and so on. Level 4 was the greatest level of syncopation, with four events on weak beats and four events on strong beats. Each pattern consisted of 8 sounded events (262 Hz sine waves, 50 ms duration, 5-ms rise and fall times) laid out on an eight-beat metrical grid with binary subdivisions. Thus, the total duration of each pattern was sixteen (eighth-note) beats, equivalent to eight quarter-note beats or two four-beat measures. Each trial consisted of six pattern repetitions. Different combinations of tempo and rhythm were presented in a pseudorandom order such that consecutive trials always had different rhythms, and different tempos. Thus, participants were forced to find both the frequency and the phase of the pulse anew for each rhythm; they could not simply tap at the same tempo throughout the experiment.

**Figure 7:**
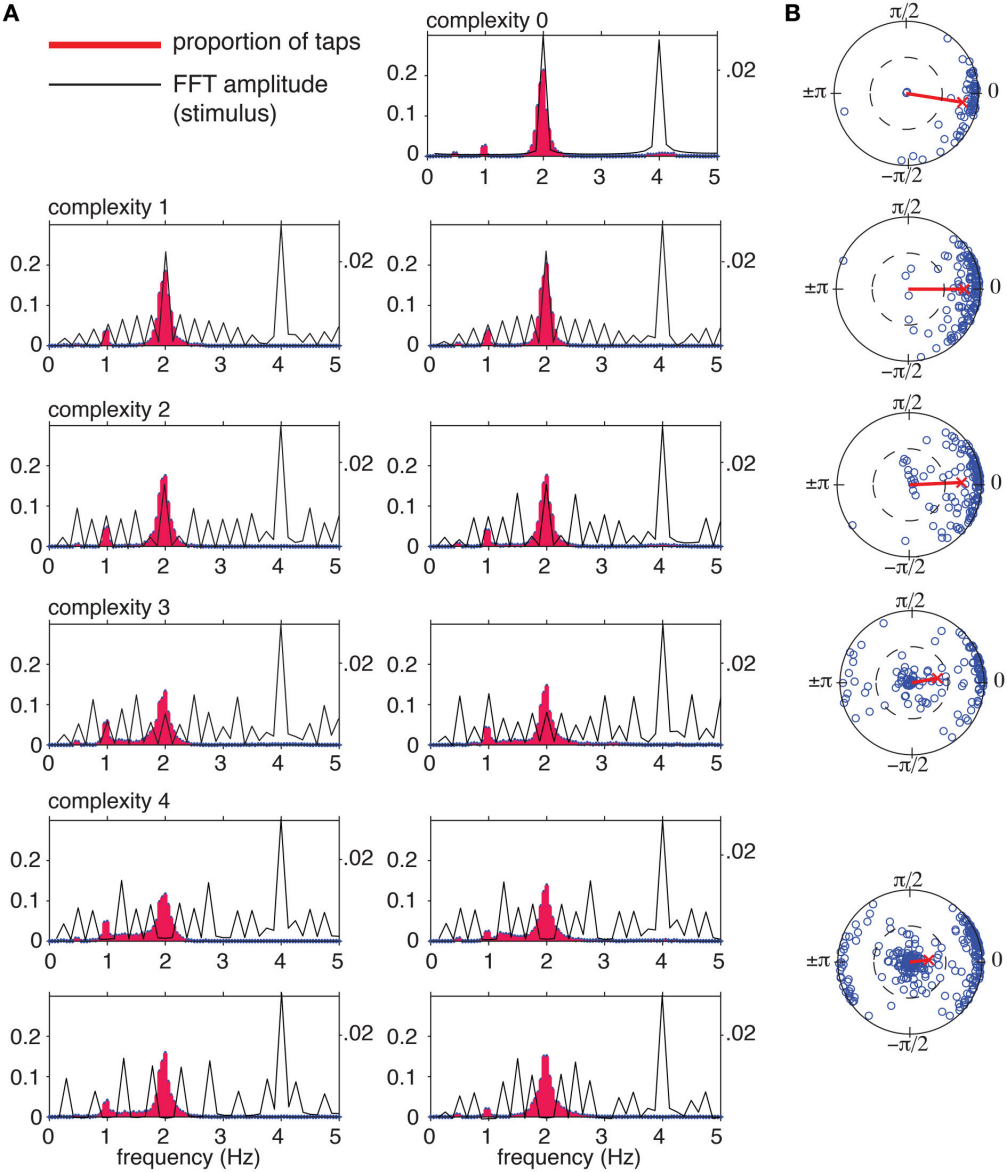
**(A)** Tapping frequencies were normalized to a 2 Hz (120 bpm) tempo to allow comparison between trials at different tempos. Tapping frequency distributions (red histograms) were computed by binning normalized instantaneous tapping frequencies from 0 to 5.00 Hz in bin widths of 0.05 Hz. Distributions were computed for each rhythm separately, including every tap interval across trials. Black lines show amplitude spectrum of the stimulus envelope for comparison. **(B)** Circular means of tap phases for each trial (blue circles) and grand mean for each complexity level (red line).

We measured instantaneous tapping frequency distributions to determine whether subjects induced a pulse at the intended frequency. Instantaneous tapping frequency was computed as 1∕*ITI* (*ITI* = inter-tap interval in seconds) and tapping frequencies were normalized to a frequency of 2 Hz so they could be combined into a single distribution at each level of complexity. Spectral analysis (DFT) of the stimulus rhythms (Figure 7A, black) shows that at the hypothetical pulse frequency amplitude decreases with increasing complexity. At complexity level 4, the amplitude is precisely zero at 2 and 1 Hz for each rhythm. Normalized instantaneous tapping frequency (Figure 7A, red histogram) displays a main peak at the normalized pulse frequency of 2 Hz for all rhythms at all levels of complexity, with lesser peaks at 1 and 0.5 Hz, and for some rhythms, a diffuse peak around 4 Hz. Thus, the participants most often tapped the predicted pulse frequency even for the most complex rhythms, which had no spectral amplitude at that frequency. The distributions for the higher levels of complexity (levels 3 and 4) were significantly different from the distributions for the lower complexity levels [Kolmogorov-Smirnov, *D*(*N* = 101) = 0.24, *p* < 0.01]. The change appeared to be due to a decrease in the proportion of taps at 2 Hz and an increase in the proportion of (unsynchronized) taps between 1 and 2 Hz.

Next, we examined synchronization for each trial. The sequence of tap times was converted into a sequence of phases relative to the predicted pulse frequency, and the circular mean was computed for each trial (Batschelet, 1981; Figure 7B; blue circles). The grand mean was then computed for each complexity level (Figure 7B, red line). The length, *r*, of the mean vector is the synchronization coefficient, a measure of tapping variability (1 corresponds to no variability, and 0 corresponds to maximum variability), and the angle, ϕ, of the mean vector indicates the relationship to the hypothetical pulse, where positive values of ϕ indicate late tapping and negative values indicate early taps, on average (Batschelet, 1981).

At the lowest level of complexity, mean vectors clustered near the unit circle, indicating a high degree of synchronization, and near a phase of 0, indicating in-phase tapping. As complexity increased, a growing number of vectors clustered near zero, indicating lack of synchronization. The percentages of trials that were synchronized at *r*>0.5 (a highly conservative criterion Fisher, 1993) were 97, 96, 89, 60, and 55% for complexity levels 0–4, respectively. Thus, the more complex rhythms were more difficult to synchronize with, however, the majority of the time participants synchronized at the predicted pulse frequency, even for the rhythms that had no stimulus energy at that frequency. Finally, for the most complex rhythms (levels 3 and 4) a significant number of mean relative phases clustered near a relative phase of π, indicating anti-phase tapping. The proportion of anti-phase tapping changed across the five levels of syncopation [χ^2^(1, *n* = 565) = 64.59, *p* < 0.001], but was equally distributed across tempos, [χ^2^(1, *n* = 565) = 3.16, *p*>0.10]. Although the stimulus tempo range was relatively small, the fact that this analysis showed that the majority of trials were well phase locked to the pulse indicates subjects were not merely tapping near a preferred tempo (e.g., 2 Hz). If this had been the case, these trials would not have shown such strong phase locking.

As predicted by the model, participants synchronized—either in-phase or anti-phase—predominantly at the missing pulse frequency. This behavior is consistent with the prediction that formation of the pulse percept arises due to entrainment of emergent neuronal oscillations. It also rules out the potential alternatives that synchronization is merely a consequence of a common rhythmic input, or that the pulse percept may arise due to linear resonance. Theoretically speaking, it is critical to distinguish the role of a common stimulus frequency from the intrinsic dynamics of a emergent oscillation (Whittington et al., 2000), and the missing pulse rhythms used here enabled us to dissociate the two. Such rhythms may also be useful in studying the development of rhythm perception and synchronization (e.g., Kirschner and Tomasello, 2009), as well as the capabilities and limitations of “beat-deaf” individuals (e.g., Phillips-Silver et al., 2011) and non-human animals (e.g., Patel et al., 2009; Cook et al., 2013; Large and Gray, 2015), as it allows us to tease apart the stimulus from any potential contribution of endogenous neural rhythms. Additionally, more thorough analysis of synchronization behavior will enable us to fit model parameters and evaluate the detailed predictions of the neurodynamic model.

Recent findings using the steady-state evoked potential (SS-EP) approach provide converging evidence for the hypothesis of nonlinear resonance in pulse perception. In one study, meter imagery elicited subharmonic resonance at metric frequencies for a stimulus with no energy at the to-be-imagined subharmonics (Nozaradan et al., 2011). This study provided direct evidence of mode-locking, however, the subharmonic resonance was not spontaneous, it was instructed. Therefore, it does not directly address the issue of spontaneous pulse perception in complex rhythms. In another study, the amplitudes of the SS-EPs at pulse and meter frequencies were shown to be selectively enhanced in syncopated rhythms even though acoustic energy was not predominant at these frequencies (Nozaradan et al., 2012). While the result of that study matched predictions for the sensory network (Large, 2010; Velasco and Large, 2011) such a finding is not inconsistent with linear resonance (Oppenheim and Schafer, 1975), because no responses were found at frequencies that were absent from the stimulus. This study demonstrated perceived pulse at frequencies that were completely absent from the stimulus rhythms.

## 5. Discussion

The hypothesis of neural resonance to musical rhythms has been supported by a great deal of behavioral and electrophysiological evidence since Jones's original dynamic attending proposal nearly forty years ago. Not only has the framework stood up to empirical testing, but it has turned out to be compatible and complementary in interesting ways with sensory-motor theory, originally proposed by Todd as an alternative conception of musical rhythm perception. It is not surprising that sensory and motor rhythms interact, but what is somewhat unexpected is the possibility that oscillatory sensory-motor interaction itself could give rise to the percepts of pulse and meter. These data do not speak to how the sensory and motor systems interact; for that, additional work will be needed. Moreover, it remains to test the theory at the level of neural population oscillations, using EEG and MEG in humans, which is, in fact, the level of prediction at which the mathematical theory operates. Nevertheless, the fundamental prediction of the theory, that pulse should be perceived in rhythms with no energy in the amplitude envelope at the pulse frequency, is supported by behavioral evidence.

The phenomenon demonstrated empirically here, the perception of a “missing pulse,” is reminiscent in some ways of the “missing fundamental” phenomenon of pitch perception (for a review, see Plack and Oxenham, 2005). If the energy at the fundamental frequency is removed from the complex spectrum of a periodic sound, the perceived pitch remains unchanged, matching the pitch of a sinusoid with the frequency of the missing fundamental (Schouten, 1938; Licklider, 1956). Both phenomena involve the perception of a frequency that is “missing” from the stimulus. The mechanisms of pitch perception are still debated by theorists (Plack and Oxenham, 2005). However, the analogous effect may indicate the involvement of a similar mechanism, i.e., neural oscillation, albeit at different time scales. For example, a model based on the oscillations of cochlear nucleus chopper cells has been proposed to account for complex pitch perception (Meddis and O'Mard, 2006). It is important to note, however, that while these phenomena are similar, they are not the same. Perceived pitch generally corresponds to the fundamental of a pitched sound, whereas the perceived pulse of a musical rhythm is not its fundamental frequency.

A fundamental aspect of neural computation that our model does not explicitly take into account is the discharge rate and the temporal codes of action potentials produced by single cells in the central nervous system. It has been suggested, for example, that oscillatory signals themselves do not carry information, but instead all information is represented in the spiking activity of cells (see e.g., Shadlen and Movshon, 1999). Recent neurophysiological experiments in monkeys have revealed that neurons in SMA dynamically encode duration and serial order elements of the produced intervals in a synchronization-continuation task (Merchant et al., 2013; Crowe et al., 2014; Merchant et al., 2015b). However, oscillations and spikes interact in important ways, and efforts are currently being made to understand how spike discharge information coordinates with local and global neural oscillatory activity (Kayser et al., 2009; Buzsáki et al., 2012; Musacchia et al., 2014). For example, the active sensing framework of Schroeder and colleagues has clear parallels with neural resonance theory that are beginning to be recognized and exploited (Henry and Herrmann, 2014; Musacchia et al., 2014). This approach describes rhythmic attention in terms of excitation-inhibition cycles tied to slow neural oscillations that can be entrained by rhythmic stimulation (Lakatos et al., 2005, 2008; Schroeder et al., 2010). Entrainment of cortical rhythms is thought to engage neurodynamic mechanisms of temporal prediction to segregate incoming information and organize spike timing (Musacchia et al., 2014). Experiments are investigating concepts of attending dynamics at the neuronal level and, in combination with the emerging study of behavioral synchronization in nonhuman animals (Patel et al., 2009; Zarco et al., 2009; Honing et al., 2012; Cook et al., 2013; Large and Gray, 2015), promises insights that are capable of linking neuronal dynamics with perception, attention and behavior.

Recently, Patel and Iversen have claimed that entrainment of neural oscillation to external rhythms is not sufficient to explain beat perception (Patel and Iversen, 2014). In their view, neural oscillation cannot account for the fact that: “*pure perception* of a musical beat (i.e., listening in the absence of overt movement) strongly engages the motor system,” and they suggest that “beat perception involves more than the passive entrainment of neural responses to sound.” They propose the *action simulation for auditory prediction* (ASAP) hypothesis as an alternative. However, these objections represent misconceptions regarding the fundamental nature of neural oscillation and the specific predictions of neural resonance theory. Here, we have provided a specific model of pulse and meter perception based on oscillatory interactions between auditory and motor systems, and we have tested the prediction that pulse perception is based on entrainment of active, endogenous neural oscillations. Our result conclusively rules out passive synchronization of neural activity with external rhythms. While the ASAP hypothesis makes predictions regarding the specific neural structures involved in the perception of pulse and meter, it makes no predictions regarding the nature of the computations that take place in these regions. By contrast, the neural resonance approach makes specific predictions about the nature of the neurodynamic processes involved in musical rhythm perception (see Large, 2008; Large and Snyder, 2009) and it explains a phenomenon that other models fail to explain. It does not make strong commitments to specific brain regions, however, and more empirical data will be necessary to understand how the various neural structures involved contribute to observed neurodynamics. Nevertheless, the theory is based on a canonical model of neural oscillation, thus the predictions hold for an entire family of dynamical systems, suggesting that it will scale as we learn more about the dynamical interactions in the striato-thalamo-cortical network underlying rhythm perception. Neural resonance theory is also consistent with Hebbian theory (Large, 2010), suggesting that it will be able to account for findings regarding plasticity and perceptual narrowing in rhythmic enculturation (e.g., Hannon and Trehub, 2005).

Additionally, Patel and Iversen have identified neural resonance theory with Darwin's idea that “The perception …of musical …rhythm is probably common to all animals, and no doubt depends on the common physiological nature of their nervous systems” (Patel and Iversen, 2014). They argue against neural resonance based on their interpretation that “[rhesus monkeys] do not perceive a beat in rhythmic auditory patterns …[and] could not learn to tap in synchrony with an auditory metronome.” However, their fundamental argument appears to be based on the assumption that if neural resonance underlies pulse perception in humans, similar networks must be present in all animals. Moreover, Patel (2014) and Fitch (2012) reason that if that is true, then all animals should be able to synchronize body movements to rhythms. As we have argued elsewhere (Large and Gray, 2015), species differences in perception-action coordination do not speak to the question of whether or not the fundamental neural processes are oscillatory. Species differences could be accommodated within a neural resonance framework, for example, simply in terms of the differences in auditory-motor coupling (see e.g., Merchant and Honing, 2013). Furthermore, to the extent that ASAP is to be identified with Patel's “vocal learning hypothesis,” recent observations of entrainment to musical rhythms in a sea lion (Cook et al., 2013), and entrainment to simpler rhythms in a chimpanzee (Hattori et al., 2013) and a bonobo (Large and Gray, 2015) are providing mounting counter evidence.

An understanding of the relationship between auditory neurophysiology, auditory population dynamics and auditory perception is an elusive goal, due to the intricate circuitry, the many structural levels involved, and the highly nonlinear nature of the neural responses. Significant theoretical advances will be necessary to understand signal processing, pattern formation, and plasticity in this complex and highly nonlinear system. Our approach draws upon the well-established theory of nonlinear dynamical systems to build novel and increasingly comprehensive models, and make predictions for physiological and behavioral experiments. This may allow us to directly link, for the first time, complex neuronal dynamics in animal models, neural population dynamics observed in human EEG and MEG, and perceptual dynamics assessed behaviorally, within an overarching theoretical framework that describes system dynamics at multiple levels of observation.

## 6. Funding

This work was supported in part by the grant AFOSR, 2012-16, “Signal Processing, Plasticity and Pattern Formation in Networks of Neural Oscillators,” FA9550-12-10388.

### Conflict of interest statement

The authors declare that the research was conducted in the absence of any commercial or financial relationships that could be construed as a potential conflict of interest.
